# Associations between childhood maltreatment and inflammatory markers

**DOI:** 10.1192/bjo.2018.80

**Published:** 2019-01-04

**Authors:** Alish B. Palmos, Stuart Watson, Tom Hughes, Andreas Finkelmeyer, R. Hamish McAllister-Williams, Nicol Ferrier, Ian M. Anderson, Rajesh Nair, Allan H. Young, Rebecca Strawbridge, Anthony J. Cleare, Raymond Chung, Souci Frissa, Laura Goodwin, Matthew Hotopf, Stephani L. Hatch, Hong Wang, David A. Collier, Sandrine Thuret, Gerome Breen, Timothy R. Powell

**Affiliations:** King's College London, Social, Genetic and Developmental Psychiatry Centre, UK; Academic Clinical Senior Lecturer, Institute of Neuroscience, Wolfson Research Centre, Newcastle University, Campus for Ageing and Vitality; and Northumberland Tyne and Wear NHS Foundation Trust, UK; Associate Medical Director for Research, Leeds and York NHS Partnership Foundation Trust, UK; Research Associate, Institute of Neuroscience, Wolfson Research Centre, Newcastle University, Campus for Ageing and Vitality, UK; Professor of Affective Disorders, Institute of Neuroscience, Wolfson Research Centre, Newcastle University, Campus for Ageing and Vitality; and Northumberland Tyne and Wear NHS Foundation Trust, UK; Emeritus Professor, Institute of Neuroscience, Wolfson Research Centre, Newcastle University, Campus for Ageing and Vitality; and Northumberland Tyne and Wear NHS Foundation Trust, UK; Honorary Professor of Psychiatry, Neuroscience and Psychiatry Unit, Manchester University and Manchester Academic Health Science Centre, UK; Associate Clinical Researcher, Consultant Psychiatrist, Institute of Neuroscience, Wolfson Research Centre, Newcastle University, Campus for Ageing and Vitality; and Northumberland Tyne and Wear NHS Foundation Trust, UK; Professor of Mood Disorders, King's College London, Psychological Medicine, Institute of Psychiatry, Psychology and Neuroscience, South London and Maudsley NHS Foundation Trust; and National Institute for Health Research Biomedical Research Centre for Mental Health, Institute of Psychiatry, Psychology and Neuroscience, the Maudsley Hospital and King's College London, UK; Postdoctoral Research Associate, King's College London, Psychological Medicine, Institute of Psychiatry, Psychology and Neuroscience; and National Institute for Health Research Biomedical Research Centre for Mental Health, Institute of Psychiatry, Psychology and Neuroscience, the Maudsley Hospital and King's College London, UK; Professor of Psychopharmacology and Affective Disorders, King's College London, Psychological Medicine, Institute of Psychiatry, Psychology and Neuroscience, South London and Maudsley NHS Foundation Trust; and National Institute for Health Research Biomedical Research Centre for Mental Health, Institute of Psychiatry, Psychology and Neuroscience, the Maudsley Hospital and King's College London, UK; Research Assistant, King's College London, Social, Genetic and Developmental Psychiatry Centre; and National Institute for Health Research Biomedical Research Centre for Mental Health, Institute of Psychiatry, Psychology and Neuroscience, the Maudsley Hospital and King's College London, UK; King’s NIHR Global Health Unit Coordinator, Health Services and Population Research, Institute of Psychiatry, Psychology and Neuroscience, King's College London, UK; Visiting Lecturer, Psychological Medicine, Institute of Psychiatry, Psychology and Neuroscience, King's College London; and Lecturer in Epidemiology, Department of Psychological Sciences, University of Liverpool, UK; Professor of General Hospital Psychiatry, Psychological Medicine, Institute of Psychiatry, Psychology and Neuroscience, King's College London, South London and Maudsley NHS Foundation Trust; and National Institute for Health Research Biomedical Research Centre for Mental Health, Institute of Psychiatry, Psychology and Neuroscience, the Maudsley Hospital and King's College London, UK; Reader in Sociology and Epidemiology, King's College London, Health Services and Population Research, Institute of Psychiatry, Psychology and Neuroscience, UK; Senior Research Scientist, Eli Lilly and Company, Lilly Corporate Center, USA; Research Fellow, Eli Lilly and Company, UK; Reader in Neuroscience and Mental Health, Department of Basic and Clinical Neuroscience, Institute of Psychiatry, Psychology and Neuroscience, King's College London, UK; Reader of Neuropsychiatric and Translational Genetics, Social, Genetic and Developmental Psychiatry Centre, King's College London; and National Institute for Health Research Biomedical Research Centre for Mental Health, Institute of Psychiatry, Psychology and Neuroscience, the Maudsley Hospital and King's College London, UK; Honorary Lecturer and Medical Research Council Postdoctoral Fellow, Social, Genetic and Developmental Psychiatry Centre, King's College London, UK

**Keywords:** Depressive disorders, inflammation, maltreatment

## Abstract

**Background:**

Childhood maltreatment is one of the strongest predictors of adulthood depression and alterations to circulating levels of inflammatory markers is one putative mechanism mediating risk or resilience.

**Aims:**

To determine the effects of childhood maltreatment on circulating levels of 41 inflammatory markers in healthy individuals and those with a major depressive disorder (MDD) diagnosis.

**Method:**

We investigated the association of childhood maltreatment with levels of 41 inflammatory markers in two groups, 164 patients with MDD and 301 controls, using multiplex electrochemiluminescence methods applied to blood serum.

**Results:**

Childhood maltreatment was not associated with altered inflammatory markers in either group after multiple testing correction. Body mass index (BMI) exerted strong effects on interleukin-6 and C-reactive protein levels in those with MDD.

**Conclusions:**

Childhood maltreatment did not exert effects on inflammatory marker levels in either the participants with MDD or the control group in our study. Our results instead highlight the more pertinent influence of BMI.

**Declaration of interest:**

D.A.C. and H.W. work for Eli Lilly Inc. R.N. has received speaker fees from Sunovion, Jansen and Lundbeck. G.B. has received consultancy fees and funding from Eli Lilly. R.H.M.-W. has received consultancy fees or has a financial relationship with AstraZeneca, Bristol-Myers Squibb, Cyberonics, Eli Lilly, Ferrer, Janssen-Cilag, Lundbeck, MyTomorrows, Otsuka, Pfizer, Pulse, Roche, Servier, SPIMACO and Sunovian. I.M.A. has received consultancy fees or has a financial relationship with Alkermes, Lundbeck, Lundbeck/Otsuka, and Servier. S.W. has sat on an advisory board for Sunovion, Allergan and has received speaker fees from Astra Zeneca. A.H.Y. has received honoraria for speaking from Astra Zeneca, Lundbeck, Eli Lilly, Sunovion; honoraria for consulting from Allergan, Livanova and Lundbeck, Sunovion, Janssen; and research grant support from Janssen. A.J.C. has received honoraria for speaking from Astra Zeneca, honoraria for consulting with Allergan, Livanova and Lundbeck and research grant support from Lundbeck.

Worldwide, an estimated 25% of adults have reported physical abuse in childhood. In the UK, the most comprehensive overview of child protection states that there were 47 008 sexual offences and 10 136 cruelty and neglect offences recorded against children under the age of 16 in 2014/15.^1^ Childhood maltreatment has been associated with a wide range of negative health consequences, psychosocial outcomes and heightened risk for psychiatric disorders; including anxiety disorder, bipolar disorder, delinquent behaviour, impaired cognitive development and particularly depression.^2^^–^^4^ Evidence suggests that numerous biological mechanisms become activated in response to maltreatment (for example epigenetic changes, telomere erosion, cortisol dysregulation, inflammation), which alone or in combination may explain the increased vulnerability to disorders such as depression, among adults who have been maltreated.^5^

## Current understanding

Immunoinflammatory activation, and an increased release of proinflammatory cytokines, is one biological mechanism associated with childhood maltreatment and an area of growing interest in psychiatry. Cytokines play an important role in brain development and affect neurogenesis, synaptic remodelling and neurotransmitter systems to produce behavioural change.^6^^,^^7^ Many studies have reported an increase in proinflammatory cytokines such as interleukin (IL)-1, IL-6 and tumour necrosis factor-alpha and increases in the acute phase protein C-reactive protein (CRP) in patients with major depressive disorder (MDD) and among those exposed to maltreatment.^8^ Whereas, others have reported protective effects of anti-inflammatory cytokines such as IL-10.^9^

## Aims

The current study investigated the association of inflammatory marker levels in response to childhood maltreatment. Among patients with MDD, we tested whether individuals who had been maltreated had specific differences in levels of inflammatory markers compared with those patients with MDD who had not experienced maltreatment. The rationale for this was to determine if individuals with MDD who had been maltreated represent those with an inflammatory subtype of depression; which could lead to differential diagnoses (for example depression with risk for inflammatory disease) and subsequently novel intervention strategies (such as anti-inflammatory adjuvants).

Among control participants, we tested whether individuals who had been maltreated had altered inflammatory marker levels, compared to those individuals who had not been maltreated. The rationale for this was to identify whether alterations to specific components of the immune system might mark MDD resilience in response to maltreatment, and therefore hint towards a novel treatment strategy.

We addressed these aims by assessing 41 inflammatory markers in a homogeneous treatment-resistant MDD cohort recruited as part of the Antiglucocorticoid augmentation (metyrapone) of antiDepressants in Depression (ADD) study (*n* = 164), and screened controls recruited as part of the South East London Community Health Study (SELCoH, *n* = 301). In the case and control groups separately, we investigated the association of inflammatory markers with the presence of childhood maltreatment.

## Method

### Participants

Peripheral blood samples used in this study were obtained by venepuncture as part of two separate UK studies. Controls were recruited as part of SELCoH and participants with MDD were recruited as part of the ADD study. Childhood maltreatment information was collected within both studies. After collection, serum from both studies were stored at −80°C until required. Participant information relating to each study is detailed in Table 1 and a description of each study is given below.

**Table 1:** Characteristics of the case and control groups

Characteristic	Case group, ADD participants	Control group, SELCoH participants
*n*	164	301
Age, years: mean (s.d.)	47.38 (9.63)	48.50 (16.14)
Gender, % men	40.9	47.5
Body mass index, mean (s.d.)	31.54 (6.87)	26.93 (5.24)
Ethnicity, %		
Black	1.8	20.9
White	94.5	75.4
Other	3.7	3.7
Current episode, *n*	164	0
On antidepressant, *n*	164	0
Current smoker, *n*	57	48
No maltreatment	56	256
Maltreatment	108	45

ADD, Antiglucocorticoid augmentation (metyrapone) of antiDepressants in Depression (ADD) study; SELCoH, South East London Community Health Study.

#### Control group

The participants in the control group were recruited as part of SELCoH, which is a study in London, UK investigating mental and physical health in the general South East London population.^10^ Participants in this study received detailed and repeated phenotypic assessments as part of three separate phases. The first phase was carried out to assess common health disorders and mental health disorders in South East London; the second phase aimed to examine the roles of historical social context and policy in shaping patterns of health inequalities; and in the third phase, a number of biological specimens were collected from a subset of participants including blood for serum separation. All phases collected information on psychiatric disorder symptoms. Control participants were defined as those who showed no MDD symptoms in any of the three phases of SELCoH, determined using the Clinical Interview Schedule-Revised,^11^ and who had no previous diagnosis of depression, determined by a self-report questionnaire.

#### Case group

The participants with MDD (case group) were recruited as part of the ADD study, which was a clinical trial that aimed to assess the efficacy of metyrapone (a cortisol synthesis inhibitor) as an adjuvant to selective serotonin reuptake inhibitors (SSRIs) in treating MDD in those previously shown not to respond to at least two forms of treatment (treatment-refractory MDD).^12^ Participants were recruited from multiple UK centres, which included Manchester, Leeds, Bradford and Newcastle.

Major depression diagnoses were defined using DSM-IV criteria^13^ and assessed using the Structured Clinical Interview for DSM Disorders research version.^14^ Further eligibility criteria required participants to have a Hamilton Rating Scale for Depression (HRSD) score greater than 18;^15^ have a Massachusetts General Hospital Treatment Resistant Depression staging score of 2–10;^16^ be currently taking an SSRI; be aged between 18 and 65; not have alcohol or drug dependence; be free of physical comorbidities (untreated hypothyroidism, disorders of steroid production, cardiac failure, angina, myocardial infarction, renal failure in the past 3 years); and not take a medication that would contraindicate metyrapone. We utilised blood serum collected during the screening phase of the study.

Mild and major depression symptoms at the time of blood collection, were differentiated using the HRSD, where HRSD scores of 18–19 were considered mild symptoms (no participants were recruited with a HRSD <18), and HRSD scores of 20 and above were considered moderate–severe symptoms, as described previously.^15^

### Childhood maltreatment measure

The presence of childhood maltreatment was determined using the Childhood Trauma Questionnaire (CTQ).^17^ CTQ data in our data-sets were positively skewed and remained non-normal even after log-transformation. Consequently, we generated ordinal mean maltreatment measures,^17^ which, because of the small numbers of individuals with severe maltreatment, we collapsed into ‘no maltreatment’ (0) and ‘maltreatment’ (mild, moderate or severe maltreatment) (1), (Table 1).

### Ethics

For the ADD study, clinical trial authorisation was given by the Medicines and Healthcare products Regulatory Agency (MHRA: EudraCT: 2009-015165-31). Ethical approval was granted by the Sunderland Local Research Ethics Committee (REC reference number 10/H0904/9). The SELCoH study received approval from the King's College London research ethics committee, reference PNM/12/13-152. Participants from both studies provided written informed consent.

### Inflammatory marker quantification

Upon use, serum was thawed at room temperature and 41 inflammatory markers were quantified simultaneously using multiplex enzyme-linked immunosorbent assay-based technology provided by the Meso Scale Discovery V-PLEX Plus Human Biomarker 40-Plex kit, and a customised human duplex kit assaying brain-derived neurotrophic factor and interferon-alpha, as described previously.^18^ Seven-point standard curves were run in duplicate on each plate in order to calculate absolute pg/mL values for the 80 samples assayed per plate, and a no-template control was used to correct for background fluorescence. Case and control groups were randomised across batches, and plates were scanned on the Mesoscale Scale Discovery Meso Quickplex SQ 120 reader at the Social, Genetic and Developmental Psychiatry Centre, Institute of Psychiatry, Psychology and Neuroscience, King's College London.

Pilot studies revealed very high intraplate (*r*>0.99) and interplate (*r*>0.97) correlations, suggesting single measurements were acceptably reliable using this methodology. Furthermore, known quantities within the standard curves used on each plate, correlated very highly with quantities predicted by fluorescence intensity (*r*>0.99).

### Statistical analysis

#### Data processing

Standard curves were used to determine absolute quantities (pg/mL) of each inflammatory marker. Absolute quantities (pg/mL) were then log-transformed to allow for parametric analyses. Subsequently, data points were removed if they exceeded plus or minus 2 standard deviations from the mean. We excluded inflammatory markers where greater than 30% of data was missing.

#### Maltreatment analyses

For the case and control groups separately, we performed linear regressions with log-protein level as the dependent variable and childhood maltreatment as the independent variable, alongside ethnicity, smoking, antidepressant use, study site (ADD study), plate/batch effects, gender, current depressive episode severity (ADD study), age and BMI as covariates. Within our analyses we applied the Bonferroni method of multiple testing correction, in order to minimise risk for false associations.

#### Sensitivity analyses

We individually tested the potential mediating/confounding effects of physical illness (type 2 diabetes, arthritis, cardiovascular disease, stroke, high blood pressure and cancer) and socioeconomic status, within the SELCoH study, where this data was available. Physical illness information was obtained via self-report. Socioeconomic status was determined based on an individual's type of employment: manual work, non-manual work, unemployed and economically inactive (such as retired, full-time parents, students or those unable to work because of disability). Based on our previous work (which included SELCoH),^18^ we also attempted to replicate the effects of BMI on levels of CRP and IL-6 (commonly shown to be associated with maltreatment and MDD) in the ADD study, using the same model as above.

## Results

### Inflammatory markers adequately detected in serum

**Fig. 1:**
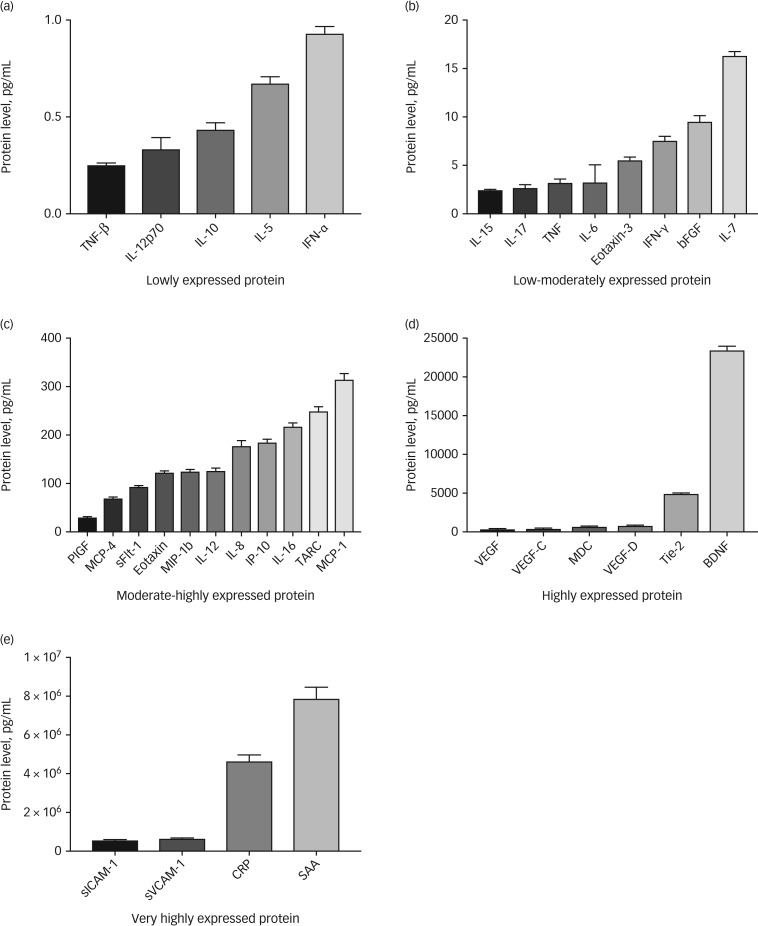
Detectable inflammatory markers. (a) Lowly expressed protein, < 1 pg/mL; (b) low–moderately expressed protein, 1 – 20 pg/mL; (c) moderate–highly expressed protein, 21 – 400 pg/mL; (d) highly expressed protein, 401 – 25,000 pg/mL; (e) very highly expressed protein, 25,001 – 100,000,000 pg/mL. Bars represent the mean and error bars represent the standard error of the mean. TNF, tumour necrosis factor; IL, interleukin; IFN, interferon; bFGF, basic fibroblast growth factor; PIGF, phosphatidylinositol glycan biosynthesis class F protein; MCP, monocyte chemoattractant protein 4; sFLT, soluble fms-like tyrosine kinase; MIP, macrophage inflammatory protein; IP, induced protein; TARC, chemokine (C-C motif) ligand 17 (also known as CCL17); VEGF, vascular endothelial growth factor; MDC, macrophage-derived chemokine; Tie, tyrosine kinases with immunoglobulin-like and EGF-like domains; BDNF, brain-derived neurotrophic factor; sICAM, soluble intercellular adhesion molecule; sVCAM, soluble vascular cell adhesion molecule; CRP, C-reactive protein; SAA, serum amyloid A.

Using our methodology, 34 inflammatory proteins passed our quality control criteria. Seven inflammatory markers were found to have greater than 30% missing data from across the whole sample and were removed from any downstream analyses (macrophage inflammatory protein-1a, granulocyte-macrophage colony-stimulating factor , IL-1a, IL-13, IL-1b, IL-2, IL-4). See Fig. 1 for a summary of detectable inflammatory markers. Known quantities within the standard curves used on all plates, correlated very highly with quantities predicted by fluorescence intensity (*r*>0.99), results also showed acceptably low levels of intraplate and interplate variability based on coefficient of variation metrics. For further details on correlations between different inflammatory markers, and assay variability metrics, see supplementary Tables 1 and 2 available at https://doi.org/10.1192/bjo.2018.80.

### Effect of maltreatment on inflammatory marker levels in case and control groups

We found no significant associations between childhood maltreatment and levels of inflammatory markers in controls (Table 2). We found one nominally significant association between maltreatment and levels of an inflammatory marker in the MDD case group, whereby, childhood maltreatment was associated with a reduction in circulating levels of serum amyloid A in adulthood (*F*(1, 143) = 4.837, *P* = 0.029, variance explained, 3.3%). This effect did not remain significant following multiple testing correction (Table 2).

**Table 2:** Analysis of associations between childhood maltreatment and levels of inflammatory markers in the case and control groups

Marker name and abbreviation	Control group				Case group			
	*F*	d.f.	*P*	Variance explained, %	*F*	d.f.	*P*	Variance explained, %
Phosphatidylinositol glycan biosynthesis class F protein (PIGF)	2.564	279	0.110	0.9	0.032	145	0.859	0.0
Tyrosine kinases with immunoglobulin-like and EGF-like domains (Tie)-2	2.705	281	0.101	1.0	1.399	147	0.239	0.9
Vascular endothelial growth factor (VEGF)	2.049	277	0.153	0.7	1.212	133	0.273	0.9
VEGF-C	0.056	283	0.813	0.0	0.001	133	0.979	0.0
VEGF-D	2.523	280	0.113	0.9	0.875	128	0.351	0.7
Basic fibroblast growth factor (bFGF)	0.003	281	0.955	0.0	0.144	122	0.705	0.1
Soluble fms-like tyrosine kinase (sFlt-1)	0.072	280	0.788	0.0	0.255	146	0.614	0.2
Eotaxin	0.594	275	0.442	0.2	0.014	142	0.906	0.0
Eotaxin-3	2.016	245	0.157	0.8	0.035	120	0.851	0.0
Induced protein (IP)-10	0.481	277	0.488	0.2	1.226	133	0.270	0.9
Monocyte chemoattractant protein (MCP)-1	2.830	283	0.094	1.0	0.022	143	0.882	0.0
MCP-4	0.955	275	0.329	0.3	0.509	137	0.477	0.4
Macrophage-derived chemokine (MDC)	0.365	276	0.546	0.1	0.024	134	0.876	0.0
Macrophage inflammatory protein (MIP)-1b	0.200	279	0.655	0.1	0.463	134	0.497	0.3
Chemokine (C-C motif) ligand 17 (TARC)^a^	0.174	282	0.677	0.1	0.812	137	0.369	0.6
Brain-derived neurotrophic factor (BDNF)	0.536	268	0.465	0.2	0.983	130	0.323	0.8
Interferon (IFN)-alpha	2.870	215	0.092	1.3	0.000	97	0.985	0.0
Interleukin (IL)-12	0.012	283	0.912	0.0	0.532	144	0.467	0.4
IL-15	0.763	281	0.383	0.3	0.137	145	0.712	0.1
IL-16	2.106	284	0.148	0.7	3.051	133	0.083	2.2
IL-17	2.039	265	0.154	0.8	0.031	140	0.861	0.0
IL-5	3.007	196	0.084	1.5	2.750	131	0.100	2.1
IL-7	1.015	284	0.314	0.4	0.141	136	0.708	0.1
Tumour necrosis factor (TNF)-beta	0.344	268	0.558	0.1	1.717	129	0.192	1.3
IFN-gamma	0.033	270	0.855	0.0	0.492	140	0.484	0.4
IL-10	0.103	265	0.749	0.0	0.230	133	0.632	0.2
IL-12p70	0.096	251	0.757	0.0	2.286	114	0.133	2.0
IL-6	0.030	265	0.864	0.0	1.693	138	0.195	1.2
IL-8	2.969	286	0.086	1.0	2.222	130	0.139	1.7
TNF	0.464	282	0.496	0.2	0.073	139	0.787	0.1
C-reactive protein (CRP)	0.002	272	0.960	0.0	3.770	141	0.054	2.6
Serum amyloid A (SAA)	1.301	266	0.255	0.5	4.837	143	0.029	3.3
Soluble intercellular adhesion molecule (slCAM)-1	1.717	272	0.191	0.6	1.363	147	0.245	0.9
Soluble vascular cell adhesion molecule (sVCAM)-1	0.530	269	0.467	0.2	0.858	148	0.356	0.6

a. Also known as CCL17.

### Sensitivity analyses

We found no significant effects of physical illness, or socioeconomic status on inflammatory markers (*P*>0.05). We replicated our previous work, showing strong positive correlations between BMI and CRP levels (*F*(1, 86) = 29.489, *P* = 5.137 × 10^–7^, variance explained, 25.5%), and between BMI and IL-6 levels (*F*(1, 85) = 32.994, *P* = 1.403 × 10^–7^, variance explained, 28%), in the ADD study.

## Discussion

### Main findings

Inflammation is one putative risk mechanism linking childhood maltreatment to adulthood depression. This study sought to investigate whether childhood maltreatment evokes differential effects on inflammatory markers among participants with MDD and controls. We studied the effects of maltreatment on 41 inflammatory markers in a UK sample of individuals with MDD and screened controls while accounting for a broad range of confounding factors.

We found no significant associations between childhood maltreatment and inflammatory markers in the control group, suggesting that inflammation may not represent a mechanism conferring resilience to MDD in response to maltreatment. Likewise, we did not find a significant association between childhood maltreatment and inflammatory markers among the case group, suggesting there may not be an inflammatory subtype of MDD related to childhood maltreatment exposure.

### Interpretation of our findings

Our negative results differ from the more common reports of higher CRP and IL-6 levels in those with a history of childhood maltreatment.^3^^,^^19^ A lack of replication here could relate to the fact that not all studies have covaried for a broad range of confounding factors as we do. For instance, studies have revealed that childhood maltreatment is associated with higher adulthood BMI, and consequently this could partially mediate previously reported associations.^20^^–^^22^ Indeed, we have recently reported major influences of BMI (as well as other factors) on inflammatory marker levels, namely CRP and IL-6, which once covaried for, removes MDD case–control differences in inflammatory marker levels.^18^ Here, we replicate the effects of BMI on CRP and IL-6 levels in the ADD study, confirming the necessity to appropriately covary for BMI and other confounders in statistical models relating to inflammatory measures.

The effect of BMI on inflammatory levels likely results from the fact that adipose tissue is known to release adipokines and proinflammatory cytokines.^23^^–^^25^ Therefore, regardless of what causes heightened inflammation among the participants with MDD or individuals who were maltreated, weight management via balanced diets and regular exercise, may be one method to reduce excessive inflammation.

### Strengths and limitations

The strengths of the current study include the fact we assessed a broad range of inflammatory proteins using validated electrochemiluminescence methods; we used a well-characterised method of assessment for childhood maltreatment subtypes; we statistically corrected for a number of confounding factors and performed appropriate sensitivity analyses; and we performed analyses in a screened control group and a homogeneous treatment-resistant MDD cohort.

However, the study also has a number of limitations that should be acknowledged. First, and foremost, our study is of a cross-sectional design capturing inflammation levels only in adulthood. A longitudinal design would allow one to determine the temporal ordering of events, and measure how maltreatment has an impact on inflammation immediately, during adolescence and then in adulthood; identifying acute and persistent effects, if any, on inflammatory markers. Second, maltreatment was captured as a binary variable and we were underpowered to assess the effects of maltreatment severity. As the majority of a individuals who had been maltreated in our two samples experienced mild–moderate maltreatment as opposed to severe maltreatment, it is possible that the biological embedding effects of stress are less penetrant in our sample, which is why we did not observe broad effects on inflammatory marker levels.

Third, all the participants with MDD were currently on antidepressant treatment, as per the recruitment criteria. As antidepressants are known to possess some anti-inflammatory properties, it is possible this may be masking the long-term effects of maltreatment on immunoinflammatory function.^26^ Fourth, the CTQ measure of childhood maltreatment is widely used and although reliable, arguably lacks validity and is subject to the biases of retrospective recall, especially in individuals with high neuroticism.^27^ Despite this, it is important to note that recent research shows a moderate–strong positive correlation between CTQ and prospective measures of maltreatment, collected as part of longitudinal studies, validating the usefulness of the CTQ as a tool for assessing maltreatment severity.^28^

Fifth, there may be other confounding factors influencing inflammatory marker levels that we were not able to include here (such as time and season of blood collection) that may have affected our results. Finally, despite representing one of the larger single studies to date assessing disorder-specific effects of maltreatment, we may still be underpowered; therefore, future studies with even larger sample sizes may be able to confirm our largely negative findings.

In conclusion, our study does not support previous research revealing associations between childhood maltreatment and inflammatory markers in either participants with MDD or controls. Instead, our findings suggest that other factors such as BMI may be more pertinent in influencing inflammatory marker levels.

## Supplementary material

For supplementary material accompanying this paper visit http://dx.doi.org/10.1192/bjo.2018.80. click here to view supplementary material
